# Determinants of Trust in Artificial Intelligence (AI) for Health-Related Decision-Making Among Adults in Saudi Arabia: A Cross-Sectional Study

**DOI:** 10.3390/healthcare14040506

**Published:** 2026-02-16

**Authors:** Bandar S. Alharbi, Majed M. Aljabri, Endale Alemayehu Ali

**Affiliations:** 1 Community and Psychiatric Mental Health Department, College of Nursing, King Saud University, Riyadh 12375, Saudi Arabia; amajad@ksu.edu.sa; 2 Department of Public Health and Primary Care, KU Leuven, Kapucijnenvoer 33, 3000 Leuven, Belgium

**Keywords:** artificial intelligence, trust, healthcare, patient satisfaction, patient–doctor relationship, mediation analysis

## Abstract

Background/Objectives: Artificial intelligence (AI) is increasingly integrated into healthcare decision-making. Public trust in AI remains a critical determinant of its acceptance and effective use. Evidence on the factors shaping trust in AI within Middle Eastern contexts, particularly Saudi Arabia, remains limited. Therefore, we aimed to identify the determinants of trust in AI for health-related decision-making and to examine a theory-informed mediation pathway in which patient satisfaction mediates the association between patient–doctor relationships and trust in AI. Methods: We conducted a cross-sectional, facility-based survey of adults in Saudi Arabia, using an electronic questionnaire distributed in four primary healthcare centers. We performed multiple linear regression to assess the association of trust in AI for health-related decision-making with patient satisfaction, patient–doctor relationships, sociodemographic characteristics, and healthcare-related factors. A mediation analysis was also employed to evaluate the indirect and direct association linking patient–doctor relationships, patient satisfaction, and trust in AI. Results: Our findings showed that patient satisfaction was positively associated with trust in AI (β = 0.54, 95% CI: 0.18–0.90), while patient–doctor relationships showed an inverse association (β = −0.34, 95% CI: −0.48 to −0.20), possibly reflecting a greater reliance on physicians’ clinical judgment and a reduced perceived need for AI-supported decision-making. Trust in AI varied across age groups, with a lower trust observed in older age categories compared with younger adults. No strong associations were observed for sex, education, body mass index, or healthcare-related factors. Patient–doctor relationship quality was indirectly associated with trust in AI via patient satisfaction (ACME = 0.138, 95% CI: 0.043–0.246), alongside a direct association with trust in AI (ADE = −0.313, 95% CI: −0.456 to −0.160). This means that patient–doctor relationships influenced trust in AI both directly and indirectly through patient satisfaction, suggesting that, while interpersonal care may reduce the reliance on AI (direct effect), enhancing patient satisfaction can partially offset this effect and promote trust in AI (indirect effect). Conclusions: These findings highlight that fostering patient-centered care and satisfaction may be crucial for promoting public trust in AI, which has important implications for AI governance, ethical deployment, and the design of AI-supported healthcare systems.

## 1. Introduction

Global healthcare is currently undergoing a fundamental shift in clinical decision-making and healthcare delivery models, driven by the rapid integration of artificial intelligence (AI) and machine learning (ML) technologies [1,2]. From predictive modeling and diagnostic imaging to telemedicine, health information services, and personalized treatment regimens, AI-driven clinical decision support systems (AI-CDSSs) offer a unique potential to improve diagnostic accuracy, reduce medical errors, and optimize clinical workflows [3,4]. But their integration raises ethical, governance, and implementation challenges [5]. The use of AI in healthcare also critically depends on the user trust of the patient, which influences whether individuals are willing to rely on, engage with, and benefit from AI technologies in healthcare [6,7].

Trust in AI is not only about how the algorithm works and its performance. Factors such as transparency, explainability, and accountability also together shape user confidence in AI recommendations [8]. It also reflects complex interactions between technology characteristics, individual user perceptions, and contextual factors within healthcare environments [6,9,10]. Previous studies highlight that transparency, explainability, and contextual fit are key determinants of whether AI tools are trusted by users, including clinicians and patients [6,11]. Complementing this evidence, recent generative AI foresight research identifies interpersonal interaction intensity as a central and reliable dimension in evaluating AI integration in medicine, suggesting that relational and human-centered aspects of care remain critical in contexts where AI is deployed. This reinforces the relevance of patient–doctor relationships in shaping trust in AI-supported health-related decision-making [12]. Empirical evidence also found a substantial variation in public and professional trust in AI. For example, trust in AI-based clinical decision support systems among healthcare workers has been shown to depend on the perceived usefulness, ease of use, and ethical considerations in implementation [11]. Recent reviews highlight that AI and machine learning can enhance noninvasive diagnostics by enabling real-time monitoring, pattern recognition, and continuous assessment across diverse modalities, while adoption remains constrained by data privacy, algorithmic fairness, regulatory challenges, and system integration, underscoring the need for patient-centered, explainable, and scalable AI solutions [13]. A broader population survey indicates that trust in the use of AI in healthcare systems is often low, particularly when patients are uncertain about responsible use in data governance frameworks [14]. A scoping study also highlighted that the perceptions and use of AI in healthcare play important roles in enhancing its adoption by various stakeholders [7]. Higher levels of institutional adoption, characterized by ethical governance, clinical integration, regulatory oversight, and accountability, have been shown to signal legitimacy and foster public trust in AI in healthcare settings [15]. A recent systematic review of 27 studies on healthcare workers’ trust in AI-based clinical decision support systems identified transparency, usability, clinical reliability, ethical safeguards, human-centered design, and training as key determinants of trust, emphasizing the importance of explainable and contextually adaptable AI tools to foster confidence among users [11].

In the Kingdom of Saudi Arabia, this digital transformation is not only a technological trend but also a strategic cornerstone of the Health Sector Transformation Program under Saudi Vision 2030. With the establishment of the Saudi Data and AI Authority (SDAIA) and the launch of the SEHA Virtual Hospital, the Kingdom has positioned itself as a regional leader in digital health [16,17]. However, a recent study revealed that only 5.9% of major hospitals in Saudi Arabia have established specialized centers for AI and 8.82% implemented AI in patient care [18].

Although previous studies have advanced the understanding of trust in AI within clinical settings, evidence on the determinants of trust in AI within Middle Eastern populations, particularly in Saudi Arabia, remains limited. Understanding and quantifying these determinants are essential given the rapid adoption of digital health technologies. Linking psychosocial factors to AI trust in non-clinical populations remains limited.

To address this gap, we aim to identify potential determinants of trust in AI for health-related decision-making among adults in Saudi Arabia. Specifically, we assess how patient satisfaction and the patient–doctor relationship relate to trust in AI, controlling for demographic and health-related covariates. These are key relational determinants through which institutional and technological factors may ultimately shape trust in AI for health-related decision-making [19,20]. Additionally, we examine whether the association of the patient–doctor relationship and AI trust is mediated by patient satisfaction, which can provide insight into the underlying mechanisms of trust formation in health technology. Elucidating these relationships will contribute to the design of patient-centered AI applications and inform strategies to foster public confidence in AI-enabled healthcare systems.

## 2. Method

### 2.1. Study Design and Setting

A cross-sectional survey was conducted among adult visitors aged 18 years and older attending four primary healthcare (PHC) centers located within Riyadh’s second and third health clusters, Saudi Arabia. These centers provide first-line healthcare services to a diverse urban population and serve as key access points for routine and preventive care.

### 2.2. Study Population and Eligibility Criteria

The study population comprised adult visitors to the selected primary healthcare (PHC) centers during the study period. Eligibility criteria included age ≥ 18 years and the ability to provide informed consent. Participants who did not complete essential sections of the questionnaire were excluded from the final sample.

### 2.3. Data Collection Procedure

Data were collected over a two-month period, from October to November 2025. The survey was administered electronically using a structured questionnaire developed in Google forms. Access to the survey was provided via a QR code printed on informed consent forms, which were displayed on bulletin boards in all four participating healthcare centers. In addition, head nurses at the selected primary healthcare centers were supplied with the consent forms and were encouraged to distribute them directly to adult visitors during routine clinical encounters. This approach was used to maximize visibility and participation while maintaining voluntary and informed consent.

### 2.4. Sampling and Participation Rate

During the data collection period, the total number of recorded adult visitors across the four participating primary healthcare centers was 5360. Of these, 625 completed the survey, 543 participants were included in the final analytical sample after excluding observations with missing data on key variables, including trust in AI, patient satisfaction (PSQ-18), patient–doctor relationships (PDRQ-9), or selected covariates, resulting in a final participation rate of 10.1%. Analyses were therefore conducted using a complete-case approach. All completed responses were included in the analysis, contingent upon the adequate reporting of key study variables. The final sample size was deemed sufficient to support the planned multivariable regression and mediation analyses. Prior methodological work indicates that mediation analysis can be reliably conducted with moderate sample sizes, particularly when effect sizes are not trivial and continuous variables are used for both the mediator and outcome [21].

### 2.5. Variable Measures

Data were collected using a structured questionnaire comprising sociodemographic characteristics, health-related behaviors, and validated psychometric scales. Sociodemographic variables included sex, age (18–25, 26–44, 45–65, ≥65 years), and education level (elementary, intermediate, high school, bachelor, master or doctorate). Health-related variables included body mass index (BMI, calculated from self-reported height and weight), chronic disease status, regular medication use, number of medical visits in the past six months (to reduce the influence of extreme values on model estimates, it was log-transformed), and current work experience in the medical field.

**Figure 1:**
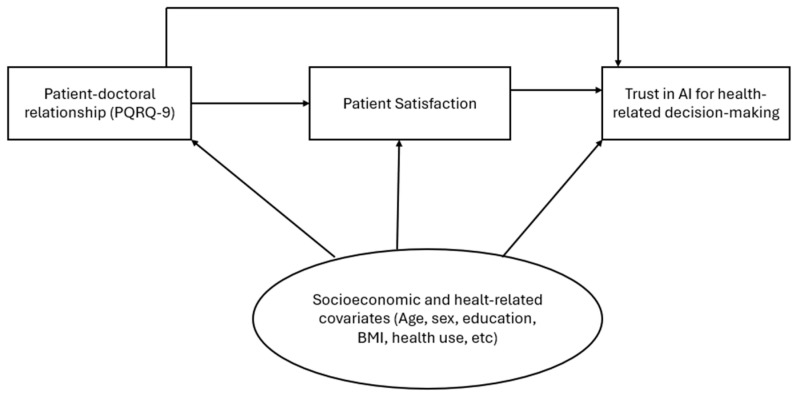
Conceptual framework of the study. The diagram illustrates hypothesized associations between patient–doctor relationship quality, patient satisfaction, and trust in AI for health-related decision-making. Sociodemographic and health-related covariates are included as potential covariates. All relationships are interpreted as associative, consistent with the cross-sectional study design.

Patient satisfaction and patient–doctor relationships were assessed using the validated Arabic versions of the Patient Satisfaction Questionnaire–18 (PSQ-18) and the Patient–Doctor Relationship Questionnaire–9 (PDRQ-9), respectively, used with permission from the original authors [22]. Negatively worded items were reverse-coded, and a composite score (PSQ_18_Total) was calculated as the mean of all item responses, with higher scores indicating greater satisfaction. A composite score (PDRQ_9_Total) was computed as the mean of the nine items, with higher values reflecting a stronger relational bond. Trust in artificial intelligence for health-related decision-making was measured using the Short Trust in Automation Scale (S-TIAS), as validated by McGrath et al. [23]. Trust in AI for health-related decision-making reflects participants’ perceived reliability and appropriateness of AI as a supportive tool in healthcare decisions, consistent with the intent of the S-TIAS. The AI trust score (AI_Trust_Total) was calculated as the mean of the three items, with higher scores indicating greater trust in AI. The conceptual framework outlining the hypothesized relationships among patient–doctor relationship quality, patient satisfaction, sociodemographic and health-related covariates, and trust in artificial intelligence for health-related decision-making is presented in Figure 1.

### 2.6. Reliability Analysis

Internal consistency reliability of all multi-item scales was assessed using Cronbach’s alpha. Reliability analyses were conducted separately for the Patient Satisfaction Questionnaire (PSQ-18), the Patient–Doctor Relationship Questionnaire (PDRQ-9), and the Artificial Intelligence Trust scale. Cronbach’s alpha coefficients, standardized alpha, and average inter-item correlations were calculated using the *psych* package in R. Values of Cronbach’s alpha ≥ 0.70 were considered indicative of acceptable internal consistency [24].

### 2.7. Statistical Analysis

Descriptive statistics were computed for all variables, with continuous data summarized as means (standard deviation) or medians (interquartile range), and categorical data as counts and percentages.

Multivariable linear regression was used to examine determinants of AI trust, with AI_Trust_Total as the dependent variable. Potential determinants included PSQ_18_Total, PDRQ_9_Total, sex, age, education, BMI, and medical visits. Categorical variables were treated as factors with explicit reference levels. Regression coefficients, standard errors, 95% confidence intervals, and *p*-values were estimated. Sensitivity analyses using robust standard errors were conducted to account for potential heteroskedasticity.

### 2.8. Model Diagnostics

Model assumptions were assessed, including the linearity, homoscedasticity, and normality of residuals. Variance inflation factors (VIF) were calculated to assess multicollinearity among predictors included in the multiple linear regression model. Generalized VIFs (GVIF) were computed for categorical variables with more than two levels, and adjusted GVIF^(1/(2 × df)) values are reported to facilitate interpretation.

### 2.9. Mediation Analysis

To investigate whether patient satisfaction mediated the relationship between patient–doctor relationships and AI trust, mediation analysis was performed using the mediation package. The mediator model regressed PSQ_18_Total onto PDRQ_9_Total and covariates including sex, age, education, BMI, chronic disease status, medication use, number of medical visits, and medical field experience. The outcome model regressed AI_Trust_Total on PSQ_18_Total, PDRQ_9_Total, and the same covariates. Nonparametric bootstrap confidence intervals (5000 resamples) were used to estimate the average mediation effect (ACME), average direct effect (ADE), total effect, and proportion mediated. Given the cross-sectional design, the terms average mediation effect, direct effect, and total effect are used to denote associational estimates rather than causal effects, and do not imply temporal or causal ordering. Factor levels were explicitly set to ensure correct interpretation, and observations with missing data in any variable were excluded.

All statistical analyses were performed with R version 4.2.1 [25].

## 3. Results

Table 1 presents the sociodemographic characteristics of the study participants. The majority of respondents were male (74%), while females accounted for 26% of the sample. Participants were predominantly young adults, with 40% aged 18–25 years, followed by those aged 45–65 years (26%) and 26–44 years (23%). Older adults aged 65 years and above represented 11% of the sample. Regarding educational attainment, nearly half of the participants held a bachelor’s degree (48%), while 30% had completed high school. Smaller proportions reported elementary (8.5%) or intermediate education (7.9%), and 5.4% had attained a master’s degree or doctorate. Missing data were observed across all sociodemographic variables (n = 83).

Table 2 summarizes the distribution of continuous study variables. The mean body mass index (BMI) of participants was 26.0 (std = 11.0), with a median of 26.0 (interquartile range [IQR]: 22–30). Participants reported a mean of 7.48 healthcare visits in the previous six months (std = 60.48); however, the median number of visits was 3.0 (IQR: 2.0–5.0).

Mean scores for patient satisfaction (PSQ-18) were high (mean = 5.67, std = 0.53; median = 5.65, IQR: 5.41–5.93). Similarly, the patient–doctor relationship score (PDRQ-9) showed a high central tendency (mean = 7.51, std = 1.35; median = 7.40, IQR: 6.80–9.00). Trust in artificial intelligence for health-related decision-making exhibited moderate to high levels (mean = 4.49, std = 1.70; median = 5.00, IQR: 3.67–5.67), with a greater variability compared to the other scales. Missing data were present for all variables.

The internal consistency reliability of the study instruments was checked using Cronbach’s α (Supplementary Table S1). The PSQ-18 demonstrated excellent reliability, with a Cronbach’s α of 0.93, indicating a high level of internal coherence among items. The standardized α yielded a comparable value (α = 0.92), suggesting that reliability was robust to item scaling. The average inter-item correlation was 0.29, which falls within the recommended range for multi-item psychological scales, indicating an adequate item homogeneity. The PDRQ-9 exhibited a very high internal consistency reliability. Cronbach’s α was 0.96, with a similar standardized alpha (α = 0.96), reflecting a strong agreement among the questionnaire items. The average inter-item correlation was 0.69, indicating a high degree of conceptual coherence among items measuring the patient–doctor relationship. AI trust also resulted in a high internal consistency, with a Cronbach’s α of 0.96 and a standardized alpha of 0.97. The average inter-item correlation was 0.87, reflecting a very strong item coherence and indicating that the scale items consistently measure a single underlying construct related to trust in AI.

Table S2 (Supplementary) presents the Pearson correlation results to examine bivariate associations between trust in artificial intelligence, patient satisfaction, and the patient–doctor relationship. No statistically significant association was observed between trust in AI and patient satisfaction (r = 0.03, 95% CI: −0.05 to 0.12, *p* = 0.446). In contrast, trust in AI was significantly and inversely correlated with the patient–doctor relationship score (r = −0.11, 95% CI: −0.19 to −0.02, *p* = 0.015). This finding suggests that individuals reporting stronger relationships with their physicians tended to express lower levels of trust in AI for health-related decision-making.

The associations between potential predictors, additional covariates, and trust in AI are presented in Table 3. We found that a higher patient satisfaction was strongly associated with a greater trust in artificial intelligence for health-related decision-making (β = 0.54, 95% CI: 0.18 to 0.90, *p* < 0.001). Conversely, a stronger patient–doctor relationship was significantly associated with a lower trust in AI (β = −0.34, 95% CI: −0.48 to −0.20, *p* < 0.001), suggesting a potential substitutive relationship between interpersonal clinical trust and reliance on AI-based tools.

A statistically significant association between age and trust in AI was observed. Compared with participants aged 18–25 years, those aged 26–44 years reported a modestly lower trust (β = −0.41, *p* = 0.04), while a substantially lower trust was observed among participants aged 45–65 years (β = −0.70, *p* < 0.001) and those aged 65 years or older (β = −1.54, *p* < 0.001). Sex showed a marginal but non-significant association, with females tending to report a lower trust in AI than males (β = −0.31, *p* = 0.07).

We did not observe significant associations between AI trust and body mass index, educational attainment, and the number of medical visits in the past six months.

We have also assessed the model diagnosis. The GVIF^(1/(2 × Df)) values ranged from 1.02 to 1.41 (Supplementary Table S3), with the highest value observed for PSQ-18_Total (1.41) and PDRQ-9_Total (1.40). Other predictors, including sex (1.07), age (1.14), BMI (1.02), education (1.08), and number of medical visits (1.03), demonstrated low GVIFs. Since all adjusted GVIFs were below the commonly accepted threshold of 2.0, no evidence of problematic multicollinearity was detected. Regression diagnostic plots, including the residuals versus fitted values, Q-Q plot, scale-location, and residuals versus leverage, indicated that the assumptions of linearity, homoscedasticity, normality of residuals, and absence of influential points were adequately met (Supplementary Figure S1).

Table 4 presents the mediation analysis. We found that the average mediation effect (ACME) was statistically significant (estimate = 0.14, 95% CI: 0.04 to 0.25, *p* = 0.008), indicating that improvements in the patient–doctor relationship were associated with an increased trust in AI indirectly through higher levels of patient satisfaction. In contrast, the average direct effect (ADE) was negative and statistically significant (estimate = −0.31, 95% CI: −0.46 to −0.16, *p* < 0.001), suggesting that, after accounting for patient satisfaction, a stronger patient–doctor relationship was directly associated with a lower trust in AI. Our finding showed that the total effect of the patient–doctor relationship on trust in AI was negative and statistically significant (estimate = −0.18, 95% CI: −0.27 to −0.07, *p* < 0.001), reflecting the combined influence of both the indirect positive association through patient satisfaction and the stronger negative direct association. The inconsistent mediation, due to the opposite directions of indirect and direct association, quantified using proportion, mediated −78.8% (95% CI: −2.03 to −0.24, *p* = 0.008). This pattern clearly showed that, while patient satisfaction increases trust in AI, a strong patient–doctor relationship independently attenuates AI trust.

## 4. Discussion

### 4.1. Main Findings

This study provides quantitative evidence on the determinants of trust in AI for health-related decision-making among adults in Saudi Arabia, highlighting the central role of healthcare experiences in shaping attitudes toward AI-supported care. Our findings indicate that trust in AI is not driven solely by technological considerations but is strongly influenced by psychosocial and relational factors embedded in routine healthcare interactions. In particular, patient satisfaction and the quality of the patient–doctor relationship emerged as key determinants, underscoring the importance of human–system interactions in the acceptance of AI-enabled health technologies. Conceptually, these findings suggest that AI trust is closely linked to existing trust structures within healthcare systems, rather than representing a distinct or independent form of trust. From a practical perspective, this implies that efforts to promote trust in AI should not focus exclusively on technical performance or explainability, but also on strengthening patient-centered care, communication, and relational continuity. In addition, the mediation analysis further indicated that patient satisfaction was associated with the relationship between the patient–doctor relationship quality and trust in AI, with evidence of both a direct association and an indirect association through patient satisfaction, suggesting a leverage point for interventions aimed at fostering public acceptance of AI-supported decision-making.

### 4.2. Comparison with Previous Studies

Several previous studies showed that patient satisfaction is a potential factor for trust in digital and AI-based health technologies. A cross-sectional survey in the United Kingdom using structured questionnaires found a positive association between healthcare experience and trust in AI-supported diagnostic tools [26]. Similarly, another study also revealed that paradox tensions are positively associated with both the intention to adopt AI systems and tools and satisfaction in healthcare services [27]. These findings align with our results, which demonstrate that patient satisfaction is significantly associated with trust in AI for health-related decision-making. This might be because satisfied patients are more likely to perceive healthcare systems as competent and patient-centered, thereby extending this trust to AI-based services for healthcare decision-making. This association emphasizes that AI trust, reinforced by clinicians and systems, is linked to the overall satisfaction with healthcare delivery [28].

According to Longoni et al. [29], patients’ resistance to AI-based medical advice is mainly driven by the neglect of uniqueness. This is because AI may be unable to account for patients’ unique personal characteristics. In line with their finding, our study also showed that the quality of the patient–doctor relationship is inversely associated with trust in AI. The possible explanation for our findings is that patients who have close communication and share decision-making with their doctor may be less likely to trust AI. Conversely, weaker patient–doctor relationships may lead to concerns that AI could depersonalize care or replace human interaction. The bivariate association between patient satisfaction and trust in AI was not statistically significant in our study. However, in the multivariable regression model, patient satisfaction showed a strong positive association with trust in AI after adjusting for patient–doctor relationship quality and other covariates. This discrepancy likely reflects the influence of confounding and suppressor effects, where the relationship between satisfaction and trust becomes more apparent once other related variables are accounted for. In particular, the patient–doctor relationship variable is inversely correlated with both satisfaction and trust in AI, which may have masked the association in the bivariate analysis.

A recent study showed that older adults expressed a greater hesitation toward AI [30]. Our finding is consistent with their finding. A qualitative study on older adults’ perspectives and acceptance of AI-driven health technologies underscored the irreplaceable role of human interaction [31]. Our findings provide limited evidence of sex differences in trust in AI, with females tending to report a lower trust in AI-based healthcare decision-making. A previous study also supported this finding. A study in China showed that males had higher odds of accepting healthcare services that used large language models [32]. Another recent study found no significant sex differences for using ChatGPT as generative AI for clinical studies and decision-making [33].

In our study, BMI, education level, and frequency of medical visits were not significantly associated with trust in AI, although most estimates suggested inverse associations. These findings suggest that general health status and healthcare utilization may not be the main determinants of trust in AI. A web-based randomized study in the USA showed that patients’ trust of AI-assisted diagnoses was consistent across age, gender, and education [34]. The non-significant finding observed in this study may, in part, be explained by the limited statistical power. On the other hand, the non-significant positive association observed among individuals with master’s and doctoral degrees may reflect that higher educational attainment facilitates the processing of complex and technical health information and supports more nuanced risk–benefit evaluations of AI-based healthcare decision-making [7].

Based on mediation analysis, our finding suggested that a strong patient–doctor relationship indirectly increases trust in AI through higher patient satisfaction, suggesting that patients with positive clinical experiences are more likely open to AI-supported healthcare decision-making [35,36]. Studies also found that patient satisfaction is a potential determinant of technology acceptance in healthcare [37,38]. On the other hand, we also found a negative significant ADE, suggesting that, once patients’ satisfaction accounted for, a strong patient–doctor relationship may directly reduce trust in AI. This finding is consistent with prior evidence [39,40]. These findings highlight the dual role of the patient–doctor relationship, whereby it may simultaneously shape trust in AI indirectly through enhanced patient satisfaction. Overall, this shows the importance of positioning AI as a supportive rather than a substitute tool in healthcare decision-making.

### 4.3. Strength and Limitations

Our study has several strengths. A main strength is its comprehensive examination of trust in AI for health-related decision-making within the Saudi Arabian context, a setting where empirical evidence on public perceptions of medical AI remains limited. By simultaneously assessing sociodemographic factors, health-related characteristics, and relational constructs such as patient–doctor relationships and patient satisfaction, the analysis provides a robust understanding of both the direct and indirect pathways shaping AI trust. The use of mediation analysis further strengthens the study by moving beyond simple associations to elucidate underlying mechanisms, thereby contributing to theory-driven evidence aligned with established models of technology acceptance and trust in automation.

Several limitations should also be acknowledged. First, the cross-sectional design precludes causal inference, particularly in the mediation analysis, as the temporal ordering between the patient–doctor relationship, satisfaction, and trust in AI cannot be definitively established. Second, the reliance on self-reported data may introduce reporting and social desirability biases, potentially affecting the accuracy of trust and satisfaction measures. Third, although the study provides valuable insights into the determinants of AI trust in Saudi Arabia, the findings may not be fully generalizable to other cultural or healthcare settings due to contextual differences in healthcare delivery, digital health adoption, and societal norms. Fourth, the sample was drawn exclusively from primary healthcare center visitors, which may overrepresent individuals already engaged with the healthcare system and limit applicability to the general population. Fifth, the study relied on a convenience sample of adults visiting primary healthcare centers, recruited via QR codes displayed on consent forms and in-person distribution. The final participation rate was modest, which may overrepresent individuals who are already engaged with the healthcare system and limit the generalizability of the findings to the broader adult population. Nonetheless, the sample included participants from multiple centers and a range of demographic backgrounds, providing meaningful insights into factors associated with trust in AI among adults actively accessing healthcare services. Moreover, the missing data required the exclusion of 82 participants from the primary regression and mediation analyses. While complete-case analysis is a standard approach, it may introduce bias if excluded participants differ systematically from those included, and it may reduce statistical power. Finally, unmeasured factors, such as prior exposure to AI technologies, digital health literacy, and specific experiences with AI-based healthcare tools—were not captured.

## 5. Conclusions

Our study provides quantitative evidence on factors associated with trust in AI for health-related decision-making. Our findings show that trust in AI is shaped by both psychosocial factors related to healthcare experiences and individual demographic characteristics, with patient satisfaction and the quality of the patient–doctor relationship emerging as central determinants. We highlight the importance of human–system interactions in shaping attitudes toward digital health technologies and identify age-related differences in AI trust. Mediation analysis further demonstrates that the association between the patient–doctor relationship quality and trust in AI operates through both direct pathways and indirect effects via patient satisfaction. From a practical perspective, AI implementation and governance strategies should integrate patient-centered care principles and clinician–patient communication to support the trust and acceptance of AI-supported decision-making. Future study using longitudinal, experimental, or mixed-methods designs is needed to validate these associations, clarify temporal relationships, and explore the underlying mechanisms shaping trust in AI-supported health-related decision-making.

## Figures and Tables

**Table 1:** Sociodemographic characteristics of the study participants (N = 625).

Characteristic	N = 625
**Sex**	n (%)
Male	401 (74%)
Female	141 (26%)
NA	83
**Age**	
18–25	217 (40%)
26–44	123 (23%)
45–65	142 (26%)
65+	60 (11%)
NA	83
**Education**	
Elementary	46 (8.5%)
Intermediate	43 (7.9%)
High school	164 (30%)
Bachelor	260 (48%)
Master or doctorate	29 (5.4%)
NA	83

**Table 2:** Descriptive statistics of continuous study variables (N = 625).

Characteristic	N = 625	
	Mean (std)	Median (IQR: Q1–Q3)
Body mass index	26 (11)	26 (22–30)
NA	82	
In the last six months, how many visits have you made?	7.48 (60.48)	3.00 (2.00–5.00)
NA	82	
PSQ_18_Total	5.67 (0.53)	5.65 (5.41–5.93)
NA	83	
PDRQ_9_Total	7.51 (1.35)	7.40 (6.80–9.00)
NA	83	
AI_Trust_Total	4.49 (1.70)	5.00 (3.67–5.67)
NA	97	

**Table 3:** Summary of multiple linear regression examining predictors of trust in artificial intelligence for health-related decision-making.

Predictor	β	95% CI	SE	*p*-Value
Intercept	5.12	[2.91, 7.33]	1.12	<0.001
PSQ_18_Total	0.54	[0.18, 0.9]	0.18	<0.001
PDRQ_9_Total	−0.34	[−0.48, −0.2]	0.07	<0.001
Sex (Female)	−0.31	[−0.65, 0.02]	0.17	0.07
Age 26–44	−0.41	[−0.8, −0.02]	0.20	0.04
Age (45–65)	−0.70	[−1.1, −0.31]	0.20	<0.001
Age (65+)	−1.54	[−2.14, −0.93]	0.31	<0.001
BMI	−0.15	[−0.6, 0.29]	0.23	0.5
Education (Intermediate)	−0.16	[−0.85, 0.53]	0.35	0.65
Education (High school)	−0.03	[−0.65, 0.59]	0.32	0.92
Education (Bachelor)	−0.06	[−0.69, 0.56]	0.32	0.84
Education (Master or doctorate)	0.19	[−0.63, 1.01]	0.42	0.65
Medical visits (log-transformed)	−0.03	[−0.06, 0]	0.02	0.09

Note: β coefficients represent adjusted associations (coefficient). Reference categories: Sex: male sex, age: 18–25 years, and education: elementary.

**Table 4:** Mediation analysis results.

Effect	Estimate	95% Confidence Interval	*p*-Value
ACME (Indirect effect)	0.138	0.043–0.246	0.008
ADE (Direct effect)	−0.313	−0.456–−0.16	<0.001
Total effect	−0.175	−0.267–−0.071	<0.001
Proportion mediated	−0.788	−2.028–−0.237	0.008

## Data Availability

Data are not shared due to privacy and ethical restrictions.

## Supplementary Materials

The following supporting information can be downloaded at: https://www.mdpi.com/article/10.3390/healthcare14040506/s1, Table S1: Internal consistency reliability of study instruments; Table S2. Pearson correlations between trust in AI, patient satisfaction, and patient–doctor relationship; Table S3. Multicollinearity diagnostics of predictors included in the multiple linear regression model (GVIF).; Figure S1: Model diagnosis.

## Abbreviations

ACME	Average Causal Mediation Effect
ADE	Average Direct Effect
AI	Artificial Intelligence
AI-CDSS	AI-Driven Clinical Decision Support Systems
BMI	Body Mass Index
CI	Confidence Interval
GVIF	Generalized Variance Inflation Factors
ML	Machine Learning
PDRQ-9	Patient–Doctor Relationship Questionnaire–9
PHC	Primary Healthcare
PSQ-18	Patient Satisfaction Questionnaire–18
SDAIA	Saudi Data and AI Authority
S-TIAS	Short Trust in Automation Scale
