# Supplementary material for: Determinants of Trust in Artificial Intelligence (AI) for Health-Related Decision-Making Among Adults in Saudi Arabia: A Cross-Sectional Study
Source: Healthcare (Basel). 2026 Feb 16;14(4):506. doi: 10.3390/healthcare14040506 (PMC12940213; doi:10.3390/healthcare14040506)
Supplement: Supplementary file 1 [file healthcare-14-00506-s001.zip › healthcare-4117425-supplementary.pdf]

Supplementary Materials

Determinants of Trust in Artificial Intelligence for Health-Related Decision-Making Among People in Saudi Arabia

Bandar S. Alharbi[1*], Majed M. Aljabri[1], Endale Alemayehu Ali[2*]

[1]Community and Psychiatric Mental Health Department, College of Nursing, King Saud University, Riyadh 12375, Saudi Arabia

[2]Department of Public Health and Primary Care, KU Leuven, Kapucijnenvoer 33, 3000 Leuven, Belgium

[*] Shared corresponding author (Email: banalharbi@ksu.edu.sa; endalestat@gmal.com)

Table S1: Internal consistency reliability of study instruments.

Scale	Items	Cronbach alpha	Standardized alpha	Average inter-item correlation
PSQ-18	18	0.92	0.92	0.29
PDRQ-9	9	0.96	0.96	0.69
Trust in AI	3	0.96	0.97	0.87

Table S2. Pearson correlations between trust in AI, patient satisfaction, and patient–doctor relationship.

Variables	r	95% CI	p-value
AI Trust—Patient Satisfaction (PSQ-18 Total)	0.03	−0.05 to 0.12	0.446
AI Trust—Patient–Doctor Relationship (PDRQ-9 Total)	−0.11	−0.19 to −0.02	0.015*

Notes: r = Pearson correlation coefficient, CI = 95% confidence interval.

Table S3. Multicollinearity diagnostics of predictors included in the multiple linear regression model (GVIF).

Predictor	GVIF	Df	GVIF^(1/(2*Df))
PSQ-18 Total	1.98	1	1.41
PDRQ-9 Total	1.97	1	1.40
Sex	1.15	1	1.07
Age	2.24	3	1.14
BMI	1.05	1	1.02
Education	1.89	4	1.08
Medical Visits	1.07	1	1.03

*Note: All GVIF^(1/(2*Df)) values < 2 indicate no problematic multicollinearity.

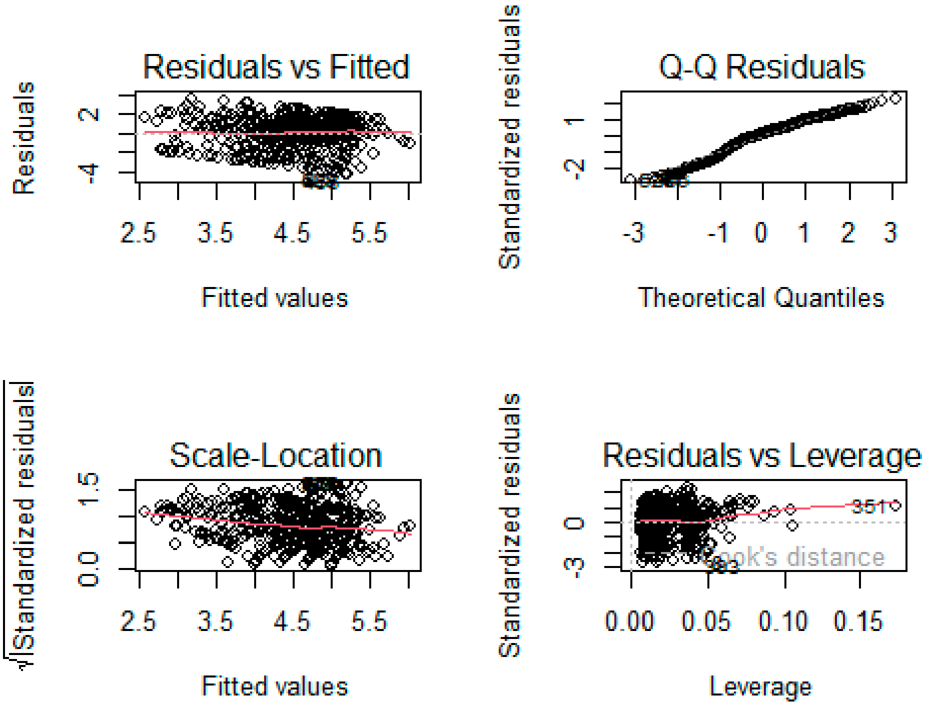

Figure S1: Model diagnosis.
